# Unisexual infection with *Schistosoma mansoni* in mice has the potential to boost the immune response against eggs after challenge infection

**DOI:** 10.3389/fimmu.2023.1125912

**Published:** 2023-02-24

**Authors:** Cindy Reinholdt, Franziska Winkelmann, Nicole Koslowski, Emil C. Reisinger, Martina Sombetzki

**Affiliations:** Department of Tropical Medicine and Infectious Diseases, Center of Internal Medicine II, Rostock University Medical Center, Rostock, Germany

**Keywords:** *Schistosoma mansoni*, long-term infection, immune response/modulation, unisexual infection, host-parasite interaction, liver fibrosis

## Abstract

**Introduction:**

The complexity of the *Schistosoma* spp. life cycle and their effective immune evasion strategies, makes vaccine development challenging. Unisexual infection models, that excludes any immunomodulatory effects of the parasite eggs, may contribute to a better understanding of complex immunological processes and identification of new targets for vaccine research. We have recently shown that long-term unisexual infection with schistosomes in mice results in an unpolarized Th1/Th2 response associated with an abnormally enlarged spleen and diffuse liver inflammation. Herein, we investigated whether (i) unisexual worms can mate after three months of single sex infection and (ii) thus the Th2 response induced by oviposition can reverse or heal the described systemic inflammation.

**Methods:**

Therefore, we infected 6–8 weeks old female C57BL/6j mice with 100 male or female cercariae and reinfected with the opposite sex for the same period after 12 weeks. At 24 weeks after initial infection, we histologically examined worm mating, as evidenced by the presence of parasite eggs, infection-related pathology associated with eggs, and characterization of fibrosis in the livers.

**Results:**

Single worms are able to mate months after unisexual infection and start oviposition. Egg deposition has been associated with a typical Th2 immune response in the liver after unisexual reinfection, accompanied by increased recruitment of CD4+ T cells. Hepatic collagen levels were significantly increased in the reinfected groups compared to the naive and unisexually infected group.

**Discussion:**

Our results indicate that the eggs are able to restore the Th1/Th2 immune balance of a previous unisexual infection. However, the organ damage caused by the unisexual worms does not subside, but rather provides the baseline for the emerging egg-triggered inflammation and fibrosis. Since single schistosomes can mate even several weeks after unisexual infection and then accumulate worm- and egg-related organ damage, infection status without positive egg detection is very important, especially in areas with low prevalence.

## 1 Introduction

Schistosomiasis, caused by dioecious trematodes of the genus *Schistosoma* spp., remains one of the most relevant helminth diseases in terms of morbidity and mortality, affecting over 230 million people worldwide and resulting in an estimated 1.6 million disability-adjusted life-years (1, 2). Three major species are responsible for the disease in humans: *Schistosoma* (*S.*) *mansoni*, *S. haematobium*, and *S. japonicum*, with most experimental studies focusing on *S. mansoni* as a representative of intestinal schistosomiasis. The causative agent of infection-associated pathology are the parasite eggs produced by the female worms after maturation and mating with males. If left untreated, infection can have serious consequences, including portal hypertension, ascites, and esophageal variceal hemorrhage leading to death. In the absence of a reliable vaccine to control the spread of the disease, current treatment strategies rely on the anthelmintic praziquantel [PZQ; (1)]. However, PZQ effectively kills adult worms, but does not provide protection against reinfection and thus is not sufficient to prevent transmission and spread in endemic areas (3, 4).

Humans as the final host are infected by floating larval stages (cercariae) of the parasite upon contact with water. These cercariae are shed by certain freshwater snails, the intermediate hosts, and find the final host by chemotactic orientation. The larvae mature into adult female and male worms during their passage through the lungs, heart, and liver, finally mate in the portal vein, where the female worms lay up to 300 eggs per day in case of *S. mansoni.* One part of the eggs penetrates the blood vessel walls, inducing granulomatous inflammation in the connective tissues and migrate into the gut lumen to be released *via* feces. The other part of the eggs is flushed back to the liver *via* bloodstream, where they become entrapped within the hepatic sinusoids and provoke a sustained inflammatory-driven process. After some years, the steadily accumulating eggs cause pronounced intestinal and hepatic fibrosis, which in turn leads to portal hypertension and its sequels - including variceal bleeding and ascites.

Paired schistosomes can survive for decades in their host, compared to unpaired worms, which were long thought to be the only ones capable of maturing and surviving, while females remain immature and die within a short period of time (5, 6). As early as 1953, experiments with rhesus monkeys showed that pre-infection with male schistosomes induces immunity against a bisexual challenge infection (7). Another study could show that the observed protective effect is indeed dependent on the sex of the parasite (8). Sex-specific differences in the induced immune response were also described in the mouse model. In unisexual infection, male worms were found to be more immunogenic than females (9). A current study demonstrated that unisexual, unpaired *S. japonicum* can survive in the host up to one year and are able to mate and produce viable eggs after that long period of time, independently if the initial infection was male or female (10). The same authors raise the question of the contribution of unisexual infections to the transmission of schistosomiasis (11). Our own studies show that a long-term unisexual infection with *S. mansoni* for one-year results in significant hepatic inflammation and tissue damage characterized by a non-polarized Th1/Th2 immune response (12). The question that remained unanswered was what happens when reinfection occurs and persistent liver inflammation caused by the worm coincides with granulomatous inflammation mediated by the eggs. Therefore, we reinfected unisexual infected mice with the opposite sex of schistosomes three months after unisexual infection and investigated whether unisexual *S. mansoni* worms can mate after several weeks and whether the thus induced oviposition and associated Th2 response can reverse the described worm-induced hepatic inflammation.

## 2 Materials and methods

### 2.1 Long-term unisexual *Schistosoma mansoni* infection of mice

*Schistosoma mansoni* (*S. mansoni*, Belo Horizonte strain) was maintained in a life cycle using *Biomphalaria glabrata* (*B. glabrata*) freshwater snails as intermediate hosts and 6-8 weeks old female NMRI mice as definitive hosts, as previously described (13). To obtain exclusively male or female cercariae for mouse infection, *B. glabrata* snails were exposed to a single *S. mansoni* miracidia. Six weeks after infection, snails were individually exposed to a strong light stimulus to obtain cercariae. The sex of the cercariae was determined by DNA amplification of sex-specific chromosome segments using female-specific primers as described previously (14). All mouse infections are performed percutaneously using the water bath method. In this way, the mice remain for about one hour in foot-high aquarium water with a defined number of cercariae. For primary infection, 6-8-weeks-old female C57BL/6 mice were infected with either 100 male-only (male, n=12) or 100 female-only (female, n=12) cercariae. A naturally, bisexually infected group, infected with a mixture of 25 male and female cercariae (control, n=12, assumed duration of egg deposition about 18-20 weeks), served as a control group. Uninfected mice served as healthy controls (naive, n=6). Other exclusively single-sex groups served as reference controls (female, n=12 or male, n=12). To investigate whether the unisexual worms are able to find each other, mate, and produce eggs even after several weeks in the unmated state, we infected unisexually again, using the opposite worm sex. Twelve weeks following primary infection, mice previously infected with 100 male cercariae were infected with 100 female cercariae (male+female, n=8, assumed duration of egg deposition about 6-8 weeks), and mice previously infected with 100 female cercariae were infected with 100 male cercariae (female+male, n=8, assumed duration of egg deposition about 6-8 weeks). Twelve weeks after the 2nd infection, mice were euthanized under ketamine/xylazine anesthesia by cervical dislocation and blood, liver and spleen were collected for further analysis.

### 2.2 Assessment of infection-related pathology

After 24 weeks, the spleen and liver weights of the mice were assessed in relation to their respective body weights. A scoring system was used to evaluate changes in the livers, taking into account changes in liver color, stiffness, and prevalence of nodules compared with naive controls (15). For histological evaluation, a piece of the right liver lobe was fixed in 4% neutral buffered formalin solution (Sigma Aldrich, Germany) and embedded in paraffin. Serial thin sections of approximately 5 μm thickness of each sample were stained with either hematoxylin/eosin (HE) or sirius red (SR). Granuloma size (HE) and SR-positive areas were evaluated using ImageJ software (v1.47v; National Institutes of Health, USA). A Quickzyme Total Collagen Assay Kit (Quickzyme Biosciences) was used to quantify the amount of total collagen in the liver based on colorimetric detection of hydroxyproline according to the manufacturer’s instructions.

### 2.3 Analysis of blood cells and serum biochemistry

At 24 weeks post-infection, mice were anesthetized with ketamine/xylazine for blood collection by puncture of the retrobulbar venous plexus. Five drops of blood were collected in a separate EDTA vial with anticoagulant, for automatic quantification of blood cells (VetScan HM5, Abaxis, USA). To determine the number of eosinophilic granulocytes, 50 µl of blood was applied to a slide and stained with Pappenheim staining. Serum biochemistry for alanine aminotransferase (ALT), aspartate aminotransferase (AST), and alkaline phosphatase was performed using the UniCel^®^ DxC 800 Synchron^®^ Clinical System (Beckman Coulter GmbH).

### 2.4 Quantitative real-time-PCR analysis

Total RNA was isolated from snap frozen liver samples (RNeasy Plus Mini Kit, Qiagen, Germany). The quantity of RNA was measured on Colibri Microvolume Spectrometer (Titertek-Berthold, Germany). 500 ng of total RNA was used to be reversely transcribed into cDNA using High-Capacity cDNA Reverse Transcriptase Kit (ThermoFisher, Germany) according to the manufacturer’s instructions. Each qRT-PCR reaction was performed using 2 µl of cDNA in a final volume of 10 µl. All samples were run in duplicates. RT-PCR was performed using the following TaqMan Gene Expression Assays: *il-1β* Mm00434228, *tnf-α* Mm00443258, *ifn-γ* Mm01168134, *il-12a* Mm00434169, *il-12b* Mm00434174_m1, *il-4* Mm00445259, *acta-2* Mm00725412, *il-13* Mm00434204, *il-10* Mm01288386 (ThermoFisher, Germany). Cycling was performed using QuantStudio 3 (Thermo Fisher Scientific, Germany) under the following reaction conditions: 50°C for 2 min followed by 95°C for 10 min, 45 cycles at 95°C for 15 s, and at 60°C for 1 min. The ΔΔCt method was utilized for relative quantification (16). Gene expression values were normalized to the endogenous reference gene GAPDH (Rodent GAPDH control reagent, ThermoFisher, Germany) and presented as normalized expression values relative to naive controls.

### 2.5 Fluorescence activated cell sorting

Single cell suspensions were prepared from whole spleens and a defined part of livers. The livers had to be digested beforehand. For this purpose, the tissue was minced and incubated in 2 ml RPMI Medium Complete (RPMI 1640 containing 10% FCS, 25 mM HEPES and penicillin/streptomycin (penicillin: 100 U/ml, streptomycin: 100 µg/ml) supplemented with 1 mg/ml Collagenase/Dispase (Sigma Aldrich, Germany) (Collagenase: 0.1 U/ml, Dispase: 0.8 U/ml) for 30 min at 37°C. After both predigested livers and spleens were passed through a cell strainer (100 µm), the tissues were washed with PBS, followed by lysis of erythrocytes with Red blood cell lysis buffer (BioLegend). Cells were washed with PBS, and cell number was quantified using a CASY TT cell counter (OLS-Omni Life Science).

A total of 10^6^ cells/sample were stained with a Zombie Red™ Fixable Viability Kit (BioLegend) for 15 min at RT in PBS followed by a 20-min incubation with mouse anti-CD16/32 (clone: 93) and stained with appropriate fluorochrome-conjugated antibodies for lymphoid lineage:: anti-CD3-APC (clone: 145-2C11), anti-CD4-PerCP-Cy5.5 (clone:RM4-4), anti-CD8-PE-Cy7 (clone: 53-6.7), anti-CD19-Alexa488 (clone: 6D5), and myeloid lineage: anti-CD11b-APC (clone: M1/70), anti-CD11c-Alexa488 (clone: N418), anti-F4/80-PE-Cy7 (clone: BM8), anti-SiglecF-PerCP (clone: E50-2440) for 20 min at 4°C in FACS Buffer (PBS with 3% FCS). Cells were then washed in PBS and fixed in 2% formaldehyde for 20 min at RT. Flow cytometric analysis was performed using the FACS Aria™ IIIu (BD Bioscience), and data were analyzed using FlowJo software (v10.0.7, Tree Star Inc., CA, USA). Live cells were differentiated by gating to the following cell populations: B cells (CD19+), T cells (CD3+), T helper cells (CD3+CD4+) cytotoxic T cells (CD3+CD8+), eosinophils (CD11b+SiglecF+), macrophages (CD11b+F4/80+), and dendritic cells (CD11c+).

### 2.6 Statistics

Statistical analysis was performed using GraphPad Prism 9.0 (GraphPad Software, La Jolla, CA, USA). Values are expressed as mean ± SEM. Data sets were compared using the Kruskal-Wallis test followed by Benjamini, Krieger and Yekutieli correction. In the context of a comparison of pathologic organ changes attributable to oviposition, all groups were examined for statistical significance compared to the unisexual groups and the naive control. In addition, with respect to comparisons in inflammatory response, the reinfection groups were also compared to the bisexually infected group. For all statistical analyses, *p* values < 0.05 were considered significant. *p < 0.05, **p < 0.01, ***p < 0.001, ****p < 0.0001.

## 3 Results

### 3.1 Unisexual *Schistosoma mansoni* long-term infection followed by challenge infection results in severe liver damage

The challenge infection with the opposite sex of *S. mansoni* was performed to analyze whether unpaired worms still find each other after several weeks, mate and start oviposition and whether the beginning oviposition can shift the unpolarized inflammation caused by the unpaired worms towards Th2 and thus reduce the diffuse inflammation.

**Figure 1:**
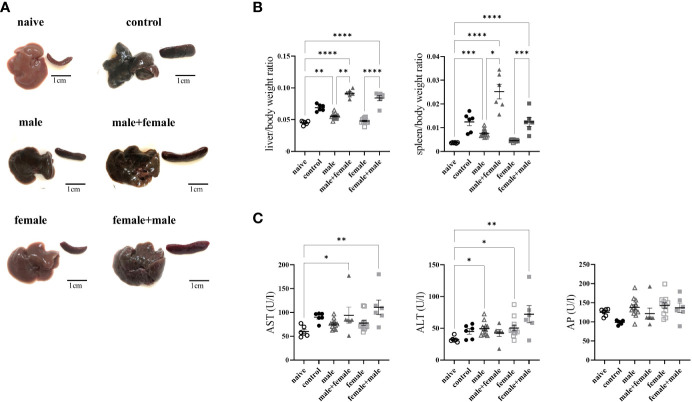
Long-term unisexual infection followed by reinfection with the opposite sex results in more severe liver damage compared with normal bisexual infection. **(A)** Representative images of livers and spleens from bisexually infected (control; infected with 25 cercariae), unisexually infected (male, female), primary unisexually infected and reinfected with the opposite sex (male+female, female+male), and naive mice 24 weeks after first infection. **(B)** Relative organ size of livers and spleens expressed as a ratio to body weight. **(C)** Serum levels of aspartate aminotransferase (AST), alanine aminotransferase (ALT) and alkaline phosphatase (AP). Data are presented as mean ± SEM. P values < 0.05 were considered statistically significant. *p < 0.05, **p < 0.01, ***p < 0.001, ****p < 0.001.

Not all mice reached the end of the experiment (control: 8 of 12; male+female: n=6 of 8; female+male: n=6 of 8). All infected groups exhibited macroscopic changes in the liver and spleen compared with naive mice, which were most pronounced in the groups with egg deposition (**Figure 1A**). Livers of male+female and female+male groups presented with grayish color and firm consistency. Hepatic granuloma formation was macroscopically visible. In the male group, livers appeared dark brown with a dull surface with no visible granulomas, while the female group had dark red colored livers with smooth surfaces and likewise with no visible granulomas. In all infected groups, the spleens were significantly enlarged (naive: 0.09 ± 0.01; control: 0.26 ± 0.08; male: 0.19 ± 0.04; female: 0.11 ± 0.01; male+female: 0.55 ± 0.14; female+male: 0.29 ± 0.1). The change in infection-associated organ alterations was determined by calculating the ratio between liver weight and body weight or spleen weight and body weight (**Figure 1B**). The liver/body weight ratio was highest in the reinfected groups male+female and female+male. The spleen/body weight ratio was significantly elevated in both reinfection groups and in the unisexually male infected group. Aspartate aminotransferase (AST), as marker for hepatocytes damage was significantly increased in all groups with oviposition (male+female, female+male) (**Figure 1C**). Mice of the female+male group and unisexually infected groups also exhibit significantly elevated alanine aminotransferase (ALT) levels, which is another marker for hepatocellular damage. However, the values of all experimental groups are within the normal range of healthy female C57BL/6 mice (AST, female C57BL/6: 193 ± 81; ALT, female C57BL/6: 95 ± 71; AP, female C57BL/6: 213 ± 44). Alkaline phosphatase, one marker of cholestasis, displayed no differences within the experimental groups.

**Figure 2:**
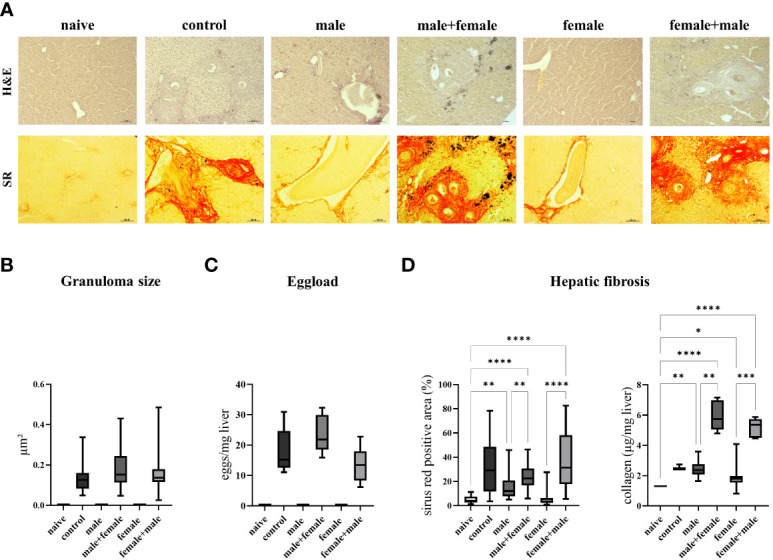
Primary infection with male schistosomes results in more severe liver fibrosis after reinfection with the opposite sex compared with normal bisexual infection. **(A)** Representative images of liver sections stained with sirius red (SR) and hematoxylin/eosin (H&E) (magnification 100-fold). Quantification of **(B)** hepatic eggs normalized to specific liver weight, granuloma diameters and **(C)** sirius red stained areas together with collagen deposition in the liver. Data are presented as mean ± SEM. P values < 0.05 were considered statistically significant. *p < 0.05, **p < 0.01 ***p < 0.001, ****p < 0.0001.

Hepatic fibrosis was quantified by histologic examination of the liver and hepatic collagen deposition. Hepatic granulomas were detected in all groups with expected oviposition, and we did not observe any differences in granuloma sizes between the groups (**Figures 2A, B**). The total number of eggs in the liver was slightly increased in the male+female group, without reaching mathematical significance, compared to the other groups with egg producing worm pairs (**Figure 2C**). The extent of liver fibrosis, represented by the sirius red-positive area, was equally increased and significantly higher in the male+female group, female+male group, and the control compared with the naive, unisexually infected groups. Moreover, the male infected group also showed a significant increase in the sirius red-positive area compared to the naive group. Furthermore, the two unisexually infected groups (male and female) showed no signs of granuloma formation despite the detection of intravascular worm segments. In a next step, liver fibrosis was quantified by measuring hydroxyproline as a marker for collagen deposition. Here, the reinfected male+female and female+male groups showed significantly higher values compared to the naive, male group, female group and the control group (**Figure 2D**). Likewise, the unisexually male infected group showed a significantly increased collagen deposition. In summary, these data demonstrate that adult worms without oviposition already trigger a mild liver fibrosis, and subsequent reinfection significantly exacerbates fibrosis, which is more severe compared with the control group with comparable hepatic egg load. This exacerbation of liver fibrosis tends to be more pronounced in the group with male primary infection than in the group with female primary infection.

### 3.2 Long-term unisexual infection with male and subsequent unisexual reinfection with female *Schistosoma mansoni* increases neutrophil and eosinophil count

**Figure 3:**
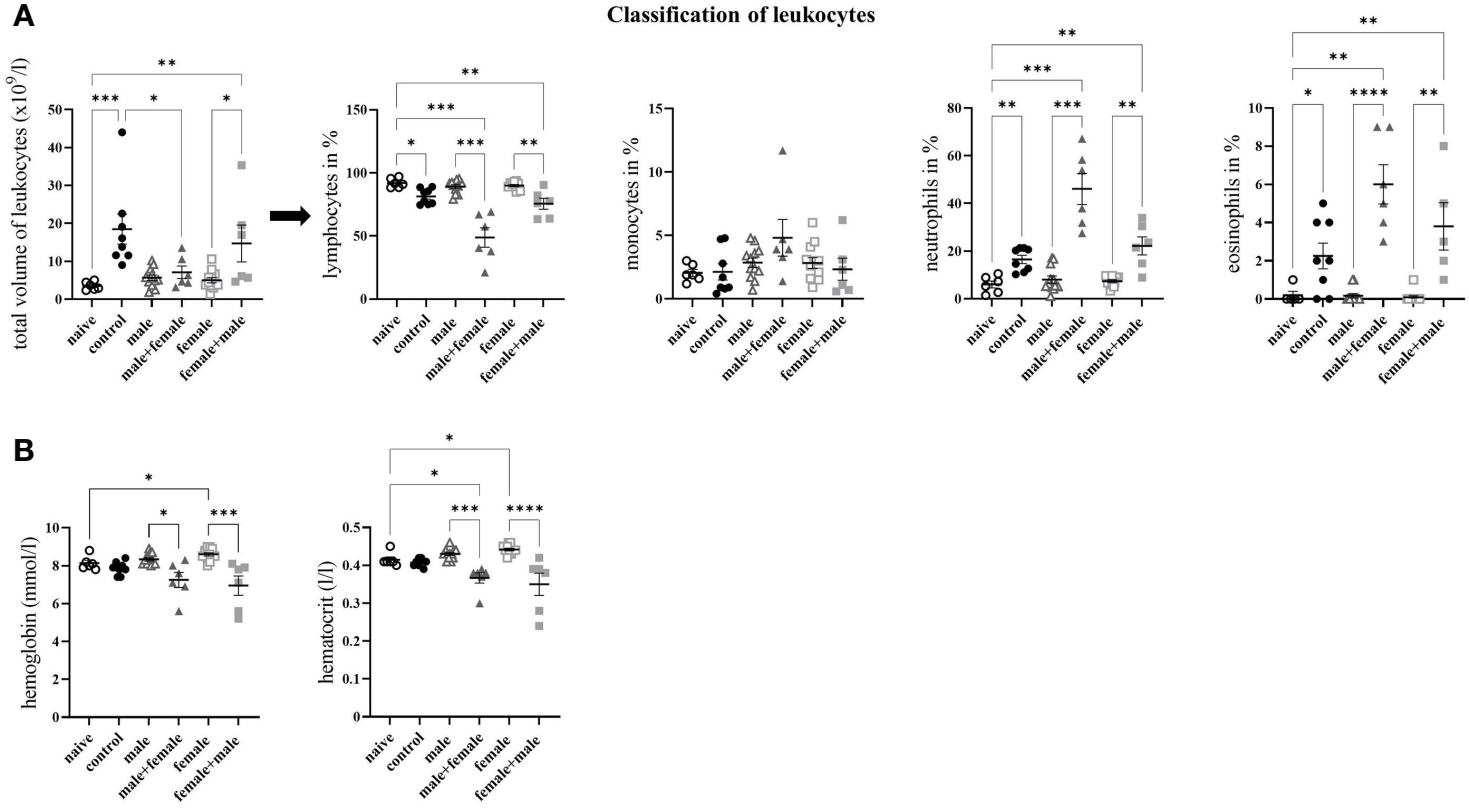
Long-term unisexual infection with male and subsequent unisexual reinfection with female *Schistosoma mansoni* increases the amount of white blood cells. **(A)** Distribution of leukocytes into lymphocytes, monocytes, neutrophils and eosinophils. **(B)** Quantification of hemoglobin concentration in blood cells and hematocrit value of blood. Data are presented as mean ± SEM. P values < 0.05 were considered statistically significant. *p* < 0.05, **p < 0.01 ***p < 0.001, ****p < 0.0001.

Because of their special function in specific and nonspecific immune defense, we determined the proportions of leukocytes in the blood of our experimental groups. In the control mice, the total number of leukocytes was significantly increased compared with the naive control group and the male+female infected group (**Figure 3A**). In the female+male group, the number of leukocytes was significantly increased in comparison to the naive control group and unisexually female infected group. To characterize the immune response more precisely, we differentiated the leukocytes and determined the percentage of lymphocytes, monocytes, neutrophils and eosinophils. Lymphocytes were significantly reduced in mice with oviposition compared with the two unisexually infected groups and the naive control group. The proportion of neutrophils and eosinophils was highest in the male+female group, and the female+male and control groups were significantly increased as well. With regard to monocytes, only in the male group was a slight increase. The unisexually infected male and female groups showed no differences in the numbers of lymphocytes, monocytes, neutrophils, and eosinophils compared with the naive control group. In addition, the hemoglobin concentration in the blood cells and the hematocrit value of the blood were examined (**Figure 3B**). In both reinfection groups (male+female, female+male), a significant decrease in hemoglobin and hematocrit was observed compared with the unisexually infected groups, whereas the control group showed only a slight decrease. Significantly increased hemoglobin and hematocrit levels were observed only in the unisex female group compared to the naive control group.

### 3.3 Unpaired schistosomes have a major impact on the upregulation of proinflammatory and profibrotic genes

**Figure 4:**
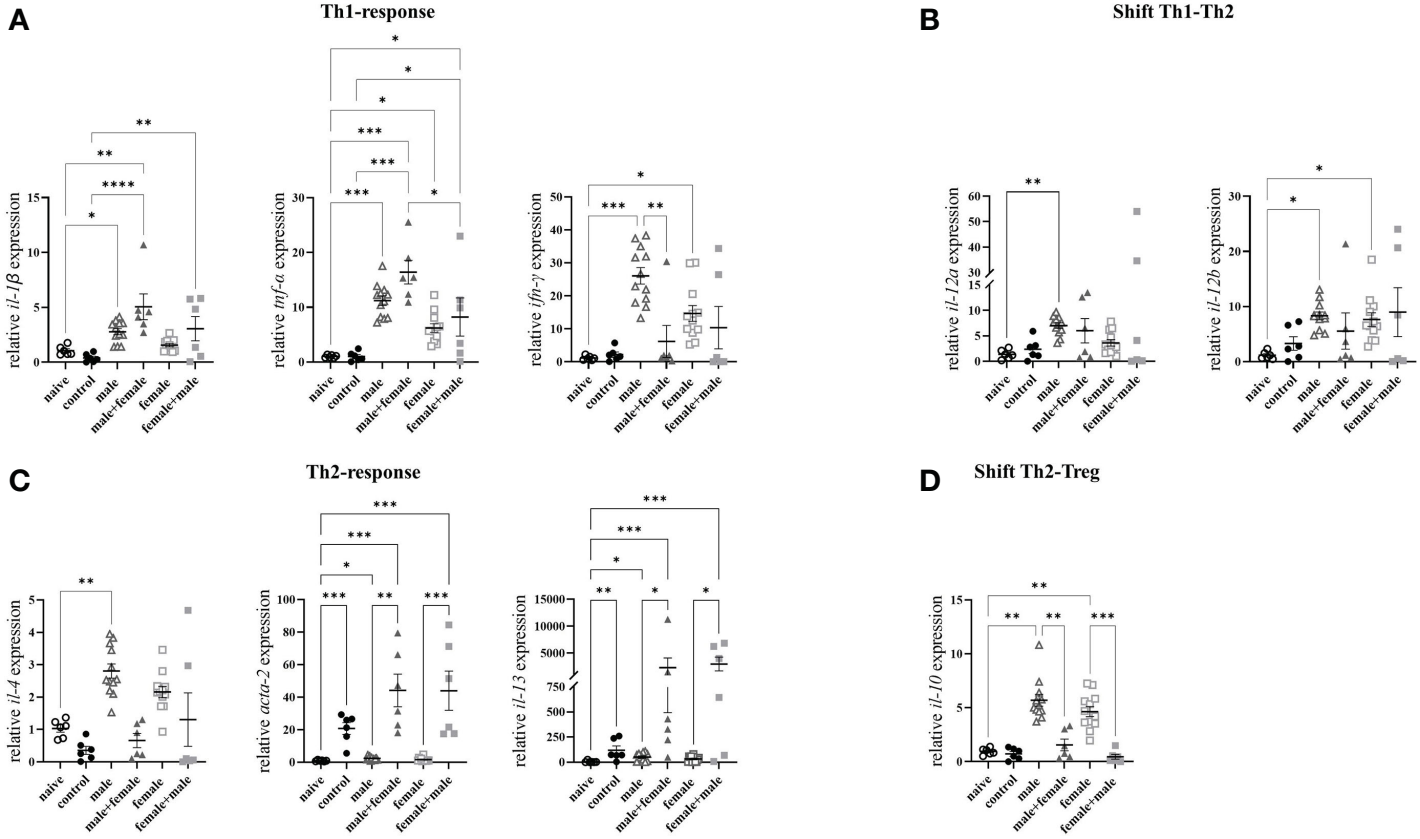
Unpaired schistosomes upregulate Th1- and Th2-associated genes, with male schistosomes having a greater effect than females. Relative expression levels of specific **(A)** Th1, **(B)** Th1-Th2-Shift, **(C)** Th2, and **(D)** Th2-Treg- Shift cytokines in livers of *Schistosoma mansoni* infected mice was determined by RT-qPCR. Data are presented as mean ± SEM. P values < 0.05 were considered statistically significant. *p < 0.05, **p < 0.01, ***p < 0.001, ****p < 0.0001.

To further characterize the hepatic inflammatory event, we quantified the expression of specific Th1- and Th2-associated genes. The Th1-associated gene interleukin (*il*)-*1β* was significantly increased in the male and male+female groups compared with the naive control group and both reinfection groups compared to the control group (**Figure 4A**). Both unisexually infected and reinfected groups showed significantly increased expression levels of *tumor necrosis factor α* (*tnf-α*). *Interferon-γ* (*ifn-γ*) was increased exclusively in the unisexually infected groups. Th1 response was not detectable in the control group. The expression levels of *il-12a* were significantly increased only in the unisexual male-infected group compared with the naive mice, whereas *il-12b* was significantly elevated in both unisexual infected groups (**Figure 4B**). Expression of *IL-4*, a typical marker of a Th2 immune response, was significantly increased only in the male group compared with the naive control group (**Figure 4C**). *Il-4* expression in the female was increased, although not significantly, compared with the naive and control groups. Expression of *alpha-actin-2* (*acta-2*) and *il-13* were significantly increased in all groups with oviposition (control, male+female and female+male). Significantly increased gene expression levels of *il-10* were observed in the unisexually infected groups without oviposition (male and female) (**Figure 4D**). In summary, unpaired schistosomes alone have a major impact on the immune response in the liver, both Th1- and Th2-associated genes are upregulated by unpaired schistosomes, with male schistosomes having a greater effect than females. After the onset of oviposition following challenge infection, fibrosis-associated genes (*il-4*, *il-13* and *acta-2*) are increasingly expressed.

### 3.4 Unisexual schistosome infection affects recruitment of inflammatory cells to spleen and liver

**Figure 5:**
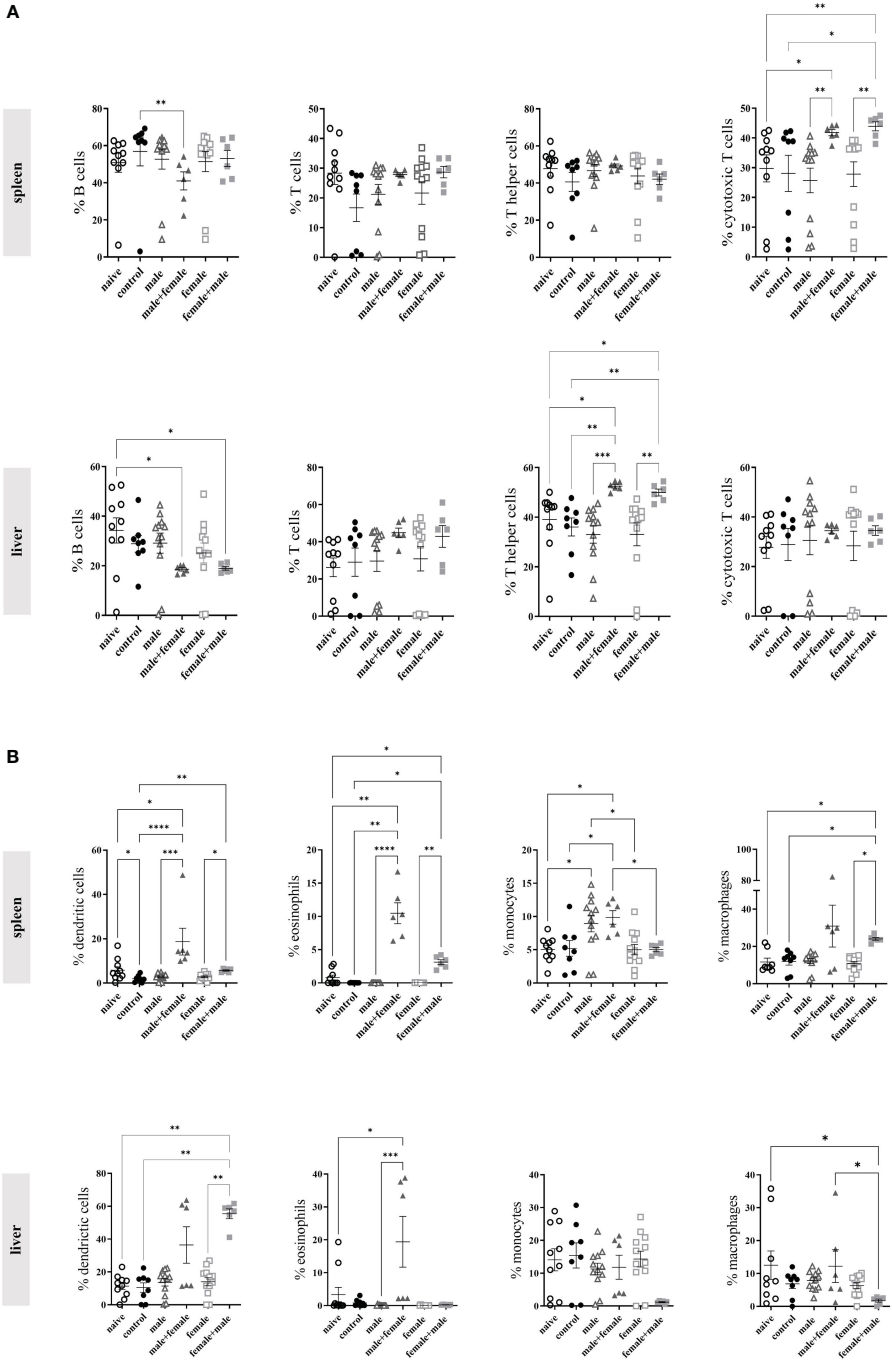
Unisexual schistosome infection increases recruitment of immune cells to the spleen and liver. **(A)** The percentage of B cells, T cells, T helper cells, cytotoxic T cells, and **(B)** dendritic cells, eosinophils, monocytes and macrophages was analyzed by flow cytometry in homogenized spleen and liver tissue from infected (control, male, male+female, female, female+male) and naive mice 24 weeks after initial infection. Data are presented as mean ± SEM. P values < 0.05 were considered statistically significant. *p < 0.05, **p < 0.01, ***p < 0.001, ****p < 0.0001.

To quantify the composition of inflammatory immune cells in the spleens and livers of our experimental animals after long-term unisexual and bisexual infection with *S. mansoni*, we performed flow cytometric analyses of fresh spleen and liver cell suspensions. In spleen cell suspensions, we observed a significant reduction of B cells in the male+female group compared with the naturally infected control group (**Figure 5A**). The percentage of T cells and T helper cells in spleens did not differ between groups, whereas significantly higher levels of cytotoxic T cells, were observed in the male+female and female+male groups. In contrast to the spleens, the livers showed significant differences in the distribution of T helper cells. Both reinfection groups, male+female and female+male, showed significantly increased numbers of T helper cells compared with all other groups. The population of B cells in the male+female and female+male groups was significantly decreased compared with the naive group. For cells of myeloid lineage, we observed significantly higher levels of dendritic cells and eosinophils in the spleens of the reinfection groups (**Figure 5B**). Both male-infected groups (male and male+female) showed a significant increase in monocytes, while the female infected group had a comparable number of monocytes as the naive mice and the control group. Only the female-male infected group had significantly increased numbers of macrophages compared with the naive, control and single-sex female infected groups.

In the myeloid cells of the liver, the percentage of dendritic cells was significantly increased only in the female+male group compared with the naive group, the control group, and the unisexually infected female group, whereas the male+female group showed only a trend toward increase. The male+female group showed a significant increase in eosinophil granulocytes. A marked decrease in macrophages was observed in the female+male group compared to the other groups. Overall, these data indicate that long-term primary infection with male or female schistosomes with subsequent reinfection results in greater recruitment of immune cells than unisexual or natural bisexual infection.

## 4 Discussion

The survivability of unisexual male and female schistosomes is controversial. It has been assumed that unisexual worms can neither survive for an extended period of time nor find a suitable mate and reproduce later (5, 17). However, several recent studies have shown that unisexual worms can survive in the host for up to a year and can mate and lay viable eggs when the opportunity arises (10, 11). Similarly, it has been hypothesized that the worms alone cause little harm to the host and only the eggs laid by female schistosomes cause lasting damage to the host. In a recently published study, we have shown that unisexual infection with *Schistosoma* (*S.*) *mansoni* over 52 weeks in a mouse model leads to extensive liver inflammation and injury associated with a non-polarized Th1/Th2 response (12). Based on these results, the question developed as to whether unpaired male or female *S. mansoni* can still mate after several weeks in the unpaired state, can begin laying eggs, and, most importantly, how disease progression occurs with the onset of egg laying. Our data presented here reveal that male and female worms can find each other and mate after a period of several weeks in the unmated state and thus start egg laying. Furthermore, we have shown that long-term unisexual infection followed by unisexual reinfection with the opposite sex results in oviposition accompanied by increased liver injury and considerably increased hepatic fibrosis compared with bisexual infection over a similar time period and comparable hepatic egg load. Regarding the extent of inflammatory reaction in livers and spleens, both reinfection groups (male+female and female+male) showed a stronger infiltration of inflammatory cells in spleens and livers compared to the respective unisexual controls or bisexually infected mice.

In humans, unisexual infections with schistosomes have received little attention. They do not elicit comparatively strong symptoms, as in the case of an asexual infection involving parasite eggs, and diagnosis is difficult because it cannot be made *via* the established egg-based parasitological tests (11). Unisexual schistosome infections in mice are highly artificial, but can provide an idea of which host immune responses are directed against the parasite eggs and which are directed against the worms. And likewise, an idea can be gained of how immunomodulatory influences are exerted by the developmental stages of the parasite, from the adult worms and which from the eggs. The crux of *Schistosoma* spp. infection is that the host is faced to different developmental stages of the parasite, from skin-penetrating cercariae, migrating larvae which pass the heart and lung, and finally egg-producing adult worm pairs which inhabit the mesenteric vasculature. Each developmental stage has its own immune evasion mechanisms, resides in different immunocompetent organs through migration, and manipulates the host in its own way to survive. There is hardly any doubt that it is mainly the eggs that are responsible for the development of a host immune response. However, studies with unisexual infection models have clearly shown that the worms themselves are effective modulators of the immune system (9, 18, 19). Furthermore, male and female schistosomes are acting differently. These differences are reflected in behavioral and physiological traits, among others: males from unisexual infections have lower body weight and are more active (20–23). Females from unisexual infections are significantly smaller and show an immature reproductive system. They require the presence of a male for complete somatic development (22, 24–26). As early as 1953, experiments with rhesus monkeys showed that pre-infection with male schistosomes induces immunity against a bisexual challenge infection (7). Sex-specific differences in the induced immune response were also described in the mouse model. In unisexual infection, male worms were found to be more immunogenic than females (9). Based on our previous studies, we can conclude that unpaired schistosomes cause an unbalanced Th1/Th2 immune response, with higher immunogenicity on the part of the male adult worms (27). This immunogenic potential of male worms was confirmed by a clinical study demonstrating Katayama syndrome, an acute inflammatory immune response, in patients as a result of infection with male *S. mansoni* (28). A clinical trial of infection with female *S. mansoni* in patients is currently ongoing (identifier ClinicalTrials.gov: NCT04269915). However, all these experimental models are artificial and can be varied in all parameters, such as infection intensity and duration, reinfection (yes or no), animal strains and others. The results may differ accordingly. In previous studies with different experimental designs, we have elaborated different and partially considerable influences of male and female worms.

In one of our first studies on this topic, we demonstrated that an initial 11 weeks lasting infection with female worms resulted in cytotoxic T-lymphocyte-associated protein 4 (CTLA-4)-mediated reduction of egg-induced hepatic fibrosis of a challenge infection. The challenge infection in this setting was bisexual (14). Male worms had no anti-fibrotic or CTLA-4 inducing effect in this setting. Belatacept, a CTLA-4 immunoglobulin, also showed anti-fibrotic effects in a subsequent study when applied before the onset of egg deposition (29). This study suggests that an anti-fibrotic effect can be achieved when T effector cell inhibition occurs and that female worms can induce this effect with an 11-week interval to reinfection. It could also be speculated that this CTLA4-mediated effect of female worms is responsible for the lower organ alterations after female-only infection compared with male-only infection. Although unpaired female worms are washed into the liver in large numbers with the bloodstream, while unpaired males remain in the portal vein, livers from mice infected with female worms are macroscopically indistinguishable from naive livers. However, another study showed that especially an initial infection with male *S. mansoni* cercariae induces a certain resistance to reinfection six weeks later. In this study, too, the challenge infection was bisexual. We utilized the air pouch model here and reinfected initially unisexually infected mice *via* the pouch and analyzed the inflammatory response to the injected cercariae. Preliminary evidence indicated an enhanced innate defense response in mice previously infected with male cercariae (27).

The main difference between the present and previous studies is the type of reinfection. We selectively reinfected with the opposite sex, which successfully resulted in egg deposition in the groups (male+female and female+male). The hemoglobin and hematocrit concentrations of the control and the groups with initiated oviposition decreased considerably and correlated directly with the egg production of the paired female worms. It has been shown that adult male schistosomes consume ~100 nl of blood per day, while females have a significantly higher consumption of ~900 nl per day (30). Blood consumption by females during unisexual infection is currently unknown, but is certainly far below that of other worms due to their smaller body size and incomplete developmental stage.

The most striking histological finding in our study is the marked enlargement of the liver and spleen after reinfection and a significant increase in liver fibrosis compared with the control group infected with both sexes. However, this comparison is not very favorable because the animals in this group were infected with only 25 cercariae. However, the hepatic egg load reached comparable levels in all three groups with egg production (control, male+female, female+male). We conclude that the estimated duration of egg laying in this group was 18-20 weeks compared to the group male+female and female+male (6-8 weeks). A comparable level of infection in the mice as in the unisexual group would be lethal for these animals during the experimental period. We therefore chose a lower intensity of infection in exchange for a long duration of infection throughout the experimental period.

Immunologically, we were able to reproduce a non-polarized Th1/Th2 response in the female and male comparison groups at the gene expression level. It could also be shown again that male schistosomes tend to have a stronger immunogenic influence than female worms (12), indicated by stronger expression of Th1 and Th2 cytokines, significantly more monocytes in the spleens and more eosinophil granulocytes in livers of the respective test animals compared to the female group. This is consistent with previous findings by and others (9, 27).

Striking are again the high *il-4* and *il-10* gene expression levels in the livers of the animals of both unisexually infected groups. IL-4 along with IL-10 are important anti-inflammatory mediators during the generation of a Th2 dominant environment protecting the host from an excessive inflammatory Th1 immune response (31, 32). Hoffman et al. performed a study using IL-10/IL-4, IL-10/IL-12 and IL-10 deficient mice to investigate the role of IL-10 in the regulation of natural *S. mansoni* infection and the effect of strictly Th1 or Th2 polarized infection on the pathology of mice (33). IL-10 was found to significantly suppress the development of type 1 and type 2 cytokines in IL-4- and IL-12-deficient mice, and thereby prevented the development of severe egg-induced pathology in the individual cytokine-deficient animals. Within the unisexually infected groups male and female, we herein have shown again a non-polarized Th1/Th2 immune response, as recently published (12). In a previous study involving the immune response to unisexual schistosome infection, splenocytes were stimulated with egg antigen and produced many more Th1 cytokines than Th-2 cytokines compared with bisexual infection (34). In addition, it has been shown that worms from unisexual infection are able to prime T helper cells and basophils to produce IL-4 and thus have an important role in the establishment of a Th2 immune milieu (35).

Flow cytometric analysis of liver cells showed significantly increased percentages of T helper cells and dendritic cells (DC) in the reinfection groups, which is appropriate because these cell types are primarily responsible for the formation of a Th2 immune milieu (36). In a study by Klaver et al., the role of *Schistosoma mansoni* soluble egg antigens (SEA) in the formation of a Th2 immune response was examined in more detail. SEA was shown to induce the expression of surface markers on DCs (PD-L1 and OX40L) known to polarize naive T cells into Th2/Treg cells. Overall, the potential of *S. mansoni* SEA glycans to modulate human DCs was demonstrated, which may contribute to the ability of SEA to down-regulate inflammatory response (37). Furthermore, the reduced B cell populations in the reinfection groups were striking in the study we present here. However, despite years of research, the function of B cells in schistosome infection is still very controversial and requires further investigation (38). Although eosinophils are highly involved in the formation of granulomas, we did not detect any increased populations except in the male+female group. We know from our own previous studies, that female schistosomes are able to suppress the innate immune system for invading cercariae, while the males trigger it (27). This might explain the lower macrophage, monocyte and also eosinophil populations in the female+male group. In contrast, we found increased macrophage and eosinophil populations in the spleen in both reinfection groups (male+female and female+male). In addition, significantly increased CD8+ cytotoxic T cells were observed in these groups. CD8+ cytotoxic T cells are typical for a Th1 immune response. Previous studies by Fallon et al. showed that apoptotic degradation of CD8+ cytotoxic T cells increases with the switch to a Th2 immune response at onset of egg deposition (39). Possible reasons mentioned were the development of the Th2 immune milieu, which mediates the degradation of Th1 cells. Alternatively, the degradation could be important to prevent type 1 inflammatory responses from exacerbating the pathology caused by the eggs. Considering this, we found elevated cytotoxic T cell levels in the spleen of the reinfection groups, which could be an indication for an incomplete switch to a Th2 immune response following onset of egg deposition. This hypothesis would also be supported by the increased Th1 cytokine levels measured in the livers of the reinfection groups.

Although the findings obtained in this study have limited applicability to humans, the results demonstrate the need for research in this area. In the future, unisexual infections with schistosomes could greatly increase. One reason for this is the reduced incidence of infected end hosts and snails as control measures take effect to contain schistosomiasis in endemic areas. As end hosts shed fewer eggs, the miracidia that hatch from them also infect fewer snails. This increases the likelihood that snails will become mono-miracidially infected, producing only one sex of cercariae that can unisexually infect the final host. This is compounded by global changes such as climate change that favor expansion of the intermediate host’s habitat. Since a 2013 outbreak of *S. haematobium* in Corsica, cases have continued to occur there (40, 41). In this context, unisexual infections with schistosomes pose some dangers that need to be considered with regard to their elimination. Conventionally, it has been assumed that unpaired male worms are capable of surviving alone for extended periods in the final host but unpaired females would starve without males (5, 6). This view has been refuted by numerous field and experimental studies in various final hosts (10, 42, 43). End hosts infected with only one sex provide a kind of “refuge” for the adult worms, where they wait for the opposite sex to successfully mate when they arrive (10). In this process, recombination of genes beneficial for survival, for example, virulence, infectivity, or drug resistance, may occur (11). Unpaired adult worms exhibit higher resistance to praziquantel than paired worms (44, 45). Hybridization of human- and livestock-pathogenic schistosome species is another problem, further facilitated by an increase in climate-induced migration (46, 47). We need to proactively address these threats by documenting the occurrence of unisexual infections with schistosomes, as well as the spread of its intermediate hosts. To do this, it is important to develop highly sensitive diagnostic tools that can be used for mass application in intermediate and definitive hosts.

## Data availability statement

The raw data supporting the conclusions of this article will be made available by the authors, without undue reservation.

## Ethics statement

The animal study was reviewed and approved by State Office for Agriculture, Food safety and Fisheries Mecklenburg-Western Pomerania (LALLF M-V/TSD/7221.3-1.1-036/18-1).

## Author contributions

CR, FW, NK, and MS designed the study. CR, FW, and MS conducted experiments and analyzed the data. CR, FW, NK, ER, and MS contributed to the data analysis. CR and MS wrote the manuscript. All authors contributed to the article and approved the submitted version.
